# Fluconazole‐induced delirium in an older patient with schizophrenia

**DOI:** 10.1111/psyg.12844

**Published:** 2022-05-23

**Authors:** Tsung‐Ming Hu, Chia‐Liang Wu

**Affiliations:** ^1^ Department of Psychiatry Taipei Veterans General Hospital, Yuli Branch Hualien Taiwan; ^2^ Department of Future Studies and LOHAS Industry Fo Guang University Jiaosi Taiwan; ^3^ Institute of Medical Sciences, Tzu Chi University Hualien Taiwan

To the editor:

Delirium is a common geriatric syndrome that is characterised by an acute change in attention, cognition, and consciousness. The common causes of delirium include medical illness, intoxication, and medications.^1^ Delirium is often treatable once the underlying aetiologies are identified and corrected. Fluconazole is effectively used for several fungal infections. The most common side effects are headache, nausea, vomiting, skin rash, and abdominal pain.^2^ Here, we describe a rare case of fluconazole‐induced delirium in an older patient with schizophrenia.

A 69‐year‐old woman was hospitalised in our psychiatric ward and presented with auditory hallucination, self‐talking, and declined cognitive function. Schizophrenia had been diagnosed when she was 35 years old. During admission, she was treated with risperidone 2 mg/day for psychotic symptoms and biperiden 4 mg/day for antipsychotic‐induced parkinsonism. One day, she complained of severe itching of the head. Physical examinations revealed a rash, scaly skin, and pustules on the scalp. Tinea capitis was suspected and she was treated with 300 mg/day of oral fluconazole.

After 1 week, she presented with confusion, slow response, forgetfulness, and disorientation. Acute delirium was diagnosed by the rapid decline in cognitive function from baseline mental function. To identify the cause of delirium, a comprehensive evaluation was performed. Complete blood count, serum electrolytes, liver and renal function tests, blood glucose, thyroid function, chest X‐ray, brain computed tomography scan, and electroencephalogram were unremarkable. She did not have a history of substance abuse or neurological disease and no other infections were found. As these were inconclusive, we examined her current medications. Fluconazole‐induced delirium was favoured as fluconazole was only recently added. We discontinued fluconazole and delirium improved after 4 days. In this case, the adverse drug reaction (ADR) probability scale (Naranjo score) was 5, which means ‘probable’ adverse reaction of fluconazole resulting in delirium.^3^

In this case, delirium developed after oral fluconazole use and the patient recovered after treatment was discontinued. Therefore, the most probable cause of delirium in this patient was the use of fluconazole. We postulated that delirium was caused in this patient due to cholinergic deficiency. Fluconazole is a potent inhibitor of the cytochrome P450 system, particularly isozymes CYP2C19, CYP3A4, and CYP2C9.^2^ Accordingly, fluconazole may inhibit metabolism and increase the concentration of any drug metabolised by these enzymes. Therefore, anticholinergic burden might increase once fluconazole is introduced. In this case, the possible mechanism of delirium is the inhibition of metabolism of biperiden (an anticholinergic drug) by fluconazole. Notably, older patients are more sensitive to the effects of anticholinergic activity because of reduction in hepatic and renal clearance of medications and increased permeability of the blood–brain barrier within the central nervous system.^4^, ^5^ Thus, they are at higher risk for anticholinergic toxicity, which is one of the pathophysiologies of delirium.

In conclusion, this case reminds us that a patient's mental status should be monitored after fluconazole administration, especially in older patients, and identifying and managing ADR promptly could benefit patient health outcomes. However, further research is required to elucidate the possible aetiology and pathogenesis of this ADR.

## FUNDING

There is no funding source.

## DISCLOSURE

The authors declare no conflicts of interest associated with this manuscript.

## ETHICAL CONSIDERATIONS

The patient described in the study provided informed consent.

## Data Availability

Data sharing is not applicable to this article as no new data were created or analyzed in this study.
